# Magneto-responsive hyaluronan hydrogel for hyperthermia and bioprinting: Magnetic, rheological properties and biocompatibility

**DOI:** 10.1063/5.0147181

**Published:** 2023-09-07

**Authors:** L. Vítková, N. Kazantseva, L. Musilová, P. Smolka, K. Valášková, K. Kocourková, M. Humeník, A. Minařík, P. Humpolíček, A. Mráček, I. Smolková

**Affiliations:** 1 Faculty of Technology, Tomas Bata University in Zlin, Vavrečkova 5669, 76001 Zlín, Czech Republic; 2 Centre of Polymer Systems, Tomas Bata University in Zlin, tř. Tomáše Bati 5678, 76001 Zlín, Czech Republic; 3 Department of Biomaterials, Faculty of Engineering Science, Universität Bayreuth, Prof.-Rüdiger-Bormann.Str. 1, 95447 Bayreuth, Germany

## Abstract

Magneto-responsive soft hydrogels are used for a number of biomedical applications, e.g., magnetic hyperthermia, drug delivery, tissue engineering, and neuromodulation. In this work, this type of hydrogel has been fabricated from hyaluronan (HA) filled with a binary system of Al_2_O_3_ nanoparticles and multicore magnetic particles (MCPs), which were obtained by clustering of superparamagnetic iron oxide FeO_*x*_ NPs. It was established that the presence of diamagnetic Al_2_O_3_ has several positive effects: it enhances the hydrogel storage modulus and long-term stability in the cell cultivation medium; prevents the magnetic interaction among the MCPs. The HA hydrogel provides rapid heating of 0.3 °C per min under exposure to low amplitude radio frequency alternating magnetic field. Furthermore, the magneto-responsive hydrogel was successfully used to encapsulate cells and extrusion-based 3D printing with 87±6% cell viability, thus providing a bio-ink. The combination of high heating efficiency, softness, cytocompatibility, and 3D printability of magnetic HA hydrogel leads to a material suitable for biomedical applications.

## I. INTRODUCTION

Magnetic hydrogels are promising materials for biomedical applications due to their ability to mimic the microstructure of extracellular matrix and strong response to external magnetic stimulus.^1^ Magnetic iron oxides (FeO_*x*_), magnetite and maghemite, are well-recognized magnetic components of magnetically responsive hydrogels.^2^ Additionally, these materials are nontoxic and degradable *in vivo* through a biotransformation mechanism, producing nontoxic side products.^3–5^ FeO_*x*_ are found to have the ability to produce significant heat in an alternating magnetic field (AMF) due to magnetization reversal.^6^ Thus, FeO_*x*_ have been examined as potential materials for thermosensitive biological applications, controlled drug delivery,^7^ thermal neuromodulation,^8^ or magnetic hyperthermia.^9^ Controlled drug delivery typically benefits from including the magnetic material as a specific polymer matrix filler, demonstrating self-healing or thermoresponsive character.^10,11^ The use of magnetic heating for neuromodulation was pioneered by Chen *et al.*, by triggering the heat-sensitive receptors of TRPV1 ion channel.^12^ This study prompted wide research of wireless neuromodulation via magnetic heating,^13–17^ and has found use in regenerative medicine and tissue engineering research.^18,19^ Inductive heating of the magnetic particles is also utilized in hyperthermia, a selective cancer treatment method in which the magnetic material is embedded in the tumor and is heated by exposure to the external AMF. Cancer cells are selectively damaged or killed at temperatures between 42–45 °C due to apoptosis and necrosis.^6,20^ However, there are strict limitations on the frequency and amplitude of AMF due to patients' safety concerns. Currently, clinically relevant AMF parameters in hyperthermia are the frequencies within 0.05–1 MHz and amplitudes ≤ 15 kA · m^−1^,^21^ considered safe for medical applications.

The potential of FeO_*x*_ largely depends on the particle size. The bulk material exhibits so-called hard magnetism and large coercivity. Lowering the material's size leads to the multidomain structure's collapse, resulting in superparamagnetic behavior at the size below 20 nm.^6^ At this state, the magnetic particles show zero coercivity and remanence magnetization due to the very low energy barrier of magnetization reversal. Therefore, a magnetic moment in superparamagnetic NPs can rotate freely toward the direction of the magnetic field without energy loss. However, superparamagnetic FeO_*x*_ NPs tend to aggregate due to interparticle magnetic interactions, leading to the so-called multicore particles (MCPs).^22,23^ These MCPs exhibit an effective magnetic moment, a combination of individual magnetic moments of the superparamagnetic NPs cores, appearing as a ferromagnetic material and producing energy loss upon magnetization reversal in AMF.^24^

One of the significant drawbacks of using single FeO_*x*_ NPs and MCPs is their tendency to aggregate and sediment in neutral pH.^6^ A possible way to enhance the particle's stability is the addition of highly charged Al_2_O_3_ NPs, increasing the system stability upon strong electrostatic repulsion.^25^ This so-called nanoparticle haloing was first described by Tohver *et al.* for mixtures of silica microspheres and hydrous zirconia NPs.^26^ Zubir *et al.* demonstrated that Al_2_O_3_ NPs ensure the stability of the binary system containing commercial magnetite NPs of the size 50–100 nm and Al_2_O_3_ NPs at pH of 6.5.^51^ Nevertheless, utilizing this approach to stabilize magnetic MCPs has been uncommon so far. This methodology appears promising to stabilize the dispersions of monodisperse magnetic MCPs in physiological pH 7.4, although the isoelectric point of FeO_*x*_ is between pH 4-5.^27^

In magnetic hyperthermia, thermally triggered drug delivery, as well as in thermal neuromodulation, the magnetic material (heat mediator), in the form of magnetic fluid or composite, should fulfill several requirements, such as biocompatibility, possibility to be delivered to the desired site, homogeneous distribution of the magnetic particles, long term retention and high heating rate in the clinically approved AMF.^6–8^ The methods of the material administration include (a) arterial injection, (b) direct injection, (c) surgical implantation, and (d) active targeting by site-specific antibodies.^28–31^ Therefore, the matrix encapsulating the magnetic material requires specific mechanical and rheological properties. The injectability of the material allows for omitting surgical procedures, lowering the potential risks and patients' discomfort.^32^ However, utilization of the water dispersions of magnetic particles in practical medical applications revealed several drawbacks, namely, the difficulty of securing the liquid system in the desired location, uniform distribution, and long-term retention.^9^ Therefore, embedding the magnetic particles in a hydrogel matrix is a favorable strategy.^33,34^ Hyaluronan (HA) based hydrogels cross-linked via Schiff base formation represent a suitable carrier.^35,36^ They are biocompatible and biodegradable due to the natural origin of HA. Moreover, the dynamic character of Schiff base cross-links allows injectability and fast recovery even in a fully cross-linked state.^37^ The material's relatively low storage modulus and high water content resemble some human soft tissue types, such as breast fat tissue,^38^ or spinal cord neural tissue.^39^

In particular, regenerative medicine and tissue engineering can benefit from precise tissue constructs providing optimal mechanical, electrochemical, and biological environments during cell cultivation and tissue growth.^40,41^ To this end, 3D printing techniques are used with increasing popularity due to unprecedented precision.^42^ Regarding tissue engineering, bioprinting appears to be a promising approach, as it involves living cells encapsulated in the printing material during the printing process. This technique can, thus, provide a highly uniform distribution of cells throughout the whole material,^44^ while post-printing cell seeding may often result in increased cell density on the surface of the scaffold, which needs to be addressed through complicated manipulation of the printed scaffold geometry.^45^ The use of magneto-responsive hydrogels in the 3D printing of scaffolds has been reported previously,^36,43^ whereas their incorporation in a bioprinting process enabled thermally triggered neuromodulation.^46^

In the presented work, we studied the effects of diamagnetic Al_2_O_3_ NPs on the magneto-structural properties of FeO_*x*_ MCPs dispersions. It was found that the Al_2_O_3_ NPs enhance the heating efficiency of the MCPs in the AMF. MCPs and MCPs with Al_2_O_3_ NPs were used to prepare the magnetic HA hydrogel that was examined as a potential mediator of inductive heating in medical applications. The effect of the Al_2_O_3_ NPs on the heating efficiency was also confirmed in the hydrogel. The favorable rheological profile of the magneto-responsive HA hydrogel allowed extrusion-based bioprinting to be performed and thus confirmed the material's potential to develop magneto-responsive bio-ink. Therefore, the elaborated HA hydrogel filled with iron oxide MCPs and Al_2_O_3_ NPs can provide cytocompatible material capable of rapid heating in AMF while securing precise scaffold engineering through additive manufacturing technologies—3D printing and bioprinting.

## II. RESULTS AND DISCUSSION

### A. Characterization of MCPs dispersions

Water dispersions of the magnetic MCPs with low polydispersity were prepared as we described previously.^24,47^ The MCPs represent dense aggregates composed of bare magnetic iron oxide NPs of 13 nm determined by TEM. The average particle size of the MCP is 85 nm at pH 2,5 determined by DLS. With the increase in pH the MCP size increases and zeta-potential decreases, leading to the sedimentation of the particles at around pH 4.5.^27^ The stability of the dispersion in the acidic medium is ensured solely due to the electrostatic repulsion between positively charged MCPs, zeta potential 45–55 mV. In order to increase the stability at higher pH Al_2_O_3_ NPs were added to the dispersion of MCPs.^51,52^ Commercially available Al_2_O_3_ NPs are of 13 nm size and form aggregates of about 250 nm average particle size.^50,51^ Therefore, a binary system composed of magnetic MCPs of 85 nm and Al_2_O_3_ aggregates of 250 nm was obtained. Al_2_O_3_ aggregates ensure the electrostatic repulsion between magnetic MCPs, preventing them from sedimentation at higher pH.^51^ MCPs being clusters of superparamagnetic particles present a unique magnetic entity, in which each core displays a magnetic moment, but the MCP behaves as if it had one collective magnetic moment. Apparently, the presence of diamagnetic Al_2_O_3_ NP aggregates are equally distributed in between the MCP shield the magnetic interaction among the MCPs. Thus, the stability of the MCPs' dispersion increases, as their tendency to aggregate is diminished.

### B. The effect of Al_2_O_3_ on induction heating of MCPs' dispersions

**FIG. 1.**
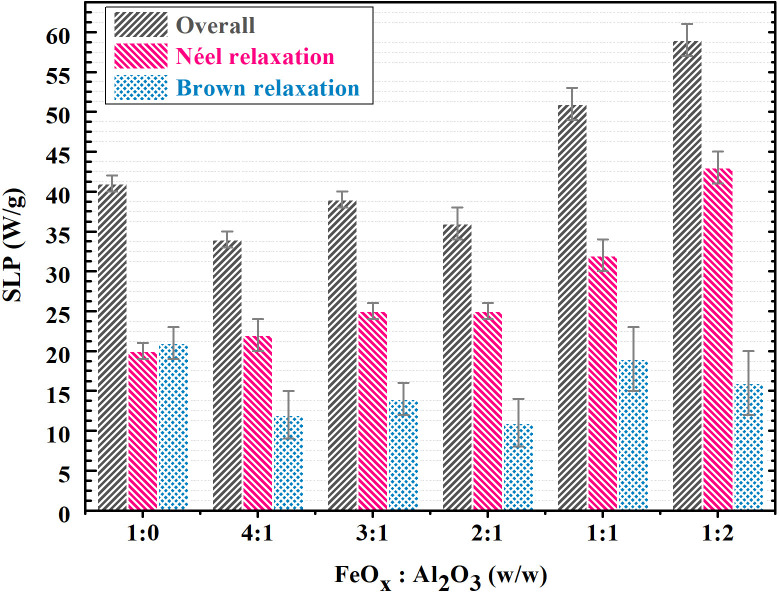
SLP of FeO_*x*_ dispersions with the increasing amount of Al_2_O_3_: overall denotes the SLP results for water dispersions, where both Brown and Néel relaxation are present; Néel relaxation denotes values obtained by measurement in agar gel, where Brown relaxation is suppressed and heating is facilitated only by Néel relaxation; Brown relaxation is the difference between overall SLP (measured in water dispersion), and Néel relaxation induced SLP (measured in agar gel).

The heating efficiency of both single MCPs and MCPs with Al_2_O_3_ aggregates was characterized by the specific loss power (SLP), i.e., the measure of the temperature change in time as referred to the mass of magnetic material. AMF of 1050 kHz, 7.4 mT was applied to the dispersions. A substantial increase from 40 W · g^−1^ for single MCPs to almost 60 W · g^−1^ for MCPs with Al_2_O_3_ aggregates was observed (Fig. 1). Due to their diamagnetic character (see Fig. S2), a direct contribution of Al_2_O_3_ NPs aggregates to the SLP is of low probability, and the increase in SLP is apparently attributed to the changes in the magnetic interactions between the MCPs.

**FIG. 2.**
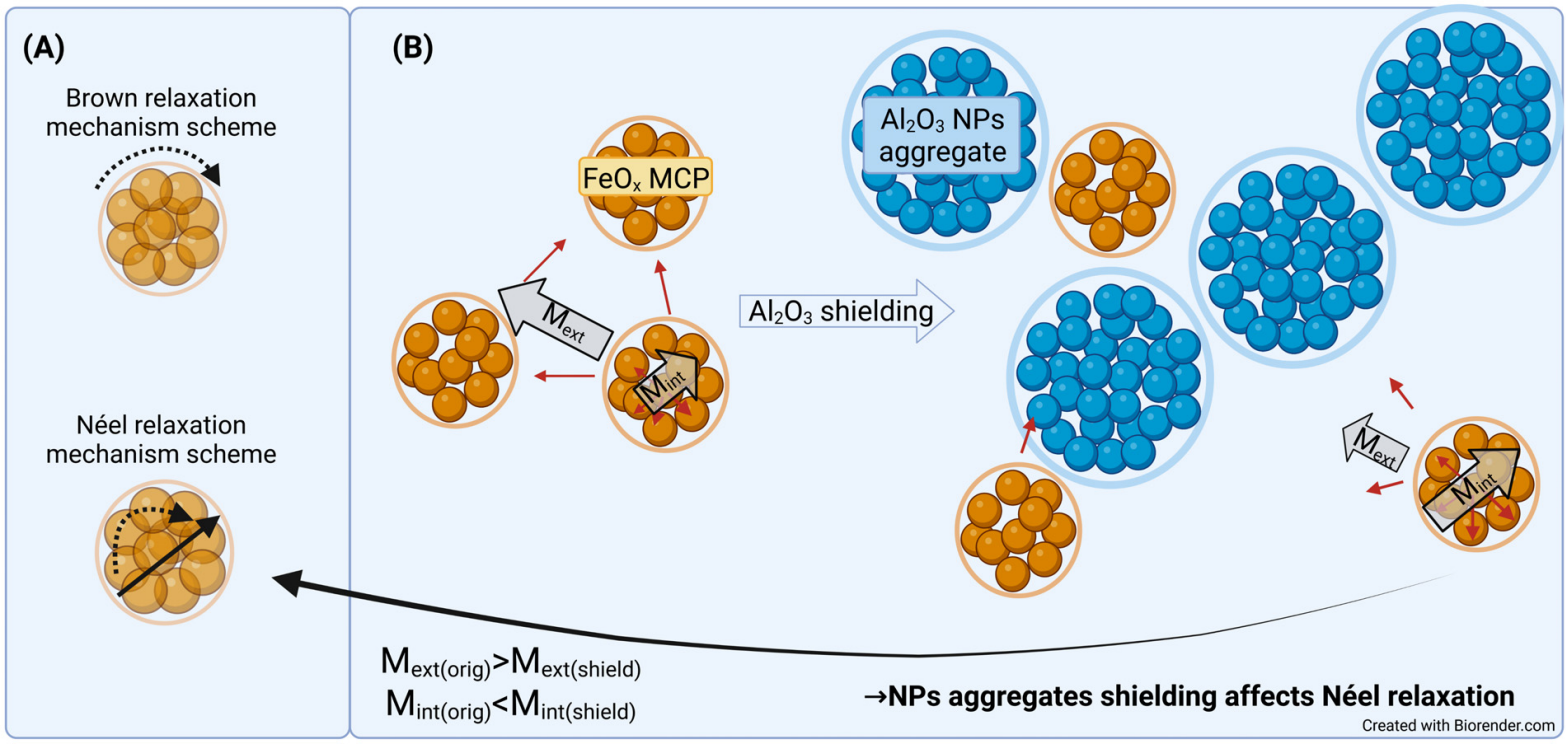
(A) Schematic representation of the relaxation mechanisms of the MCPs; and (B) schematic representation of the Al_2_O_3_ shielding effect on the magneto-structural properties of the MCPs, where M_ext_ and M_int_ represent extrinsic and intrinsic magnetic interactions of MCPs, respectively.

In general, inductive heating of the magnetic particles is the result of two relaxation processes—Brown relaxation and Néel relaxation.^53^ Brown relaxation is the process of particle rotation in the direction of the magnetic field [Fig. 2(A)].^54^ Therefore, this type of relaxation is particularly dependent on the magnetic particles volume, and on the viscosity of the dispersion medium.

$$\tau_{B}=\frac{3V_{h}\eta }{k_{B}T}.$$ (1)

Equation (1) defines the Brown relaxation time, i.e., the time necessary for the relaxation mechanism to align the magnetic moment with the direction a magnetic field. V_*h*_ denotes particle hydrodynamically effective volume, *η* is the dynamic viscosity of the dispersion medium, k_*B*_ stands for the Boltzmann constant and T is the temperature.^6,54^

Néel relaxation, on the other hand, is related to the rotation of magnetic moment within the particle, and the heating is facilitated by energy release due to the change of magnetic state of the particle. The characteristic Néel relaxation time can be found as

$$\tau_{N}=\tau_{0}e^{\frac{KV}{k_{B}T}},$$ (2)

where K is the magnetic anisotropy, V is the particle volume, k_*B*_ stands for the Boltzmann constant, T is the temperature and *τ*_0_ is the factor approximately equal to 10^−9^ s.^6,54^ It is apparent, that Néel relaxation depends only on the intrinsic properties of the magnetic particles, while Brown relaxation is largely affected by the viscosity of the used medium.^55^ In order to understand the effect of Al_2_O_3_ aggregates shielding on the induction heating behavior of the MCPs, the contributions of the two types of relaxation were examined separately. To achieve that, the SLP measurements were conducted in two dispersion media—low viscosity (demineralized water), and high viscosity (agar gel). In low viscosity medium, both relaxation mechanisms are contributing to the induction heating. However, the high viscosity of the medium restricts Brown relaxation, allowing solely the Néel relaxation.^56^ Therefore, it is possible to distinguish the contribution of Brown relaxation to the overall SLP by subtracting the Néel relaxation contribution obtained from measurement in high viscosity medium.^53^ Agar gel was chosen in this case due to exceptionally high viscosity among water-based gels (10^4^–10^5^ Pa · s depending on the concentration and shear rate^57^) that remains stable in the temperature range of the measurement (25–45 °C) in order to ensure complete prevention of the Brown relaxation driven heating.^58^

As for the Brown relaxation, the increase in Al_2_O_3_ content did not provide any significant change. Néel relaxation, on the other hand, follows the trend of overall SLP and increases with the increase in Al_2_O_3_ content. Therefore, Al_2_O_3_ shielding apparently leads to the weakening of the magnetic interactions among MCPs (Fig. 2).

### C. Morphological analysis of the hydrogels

**FIG. 3.**
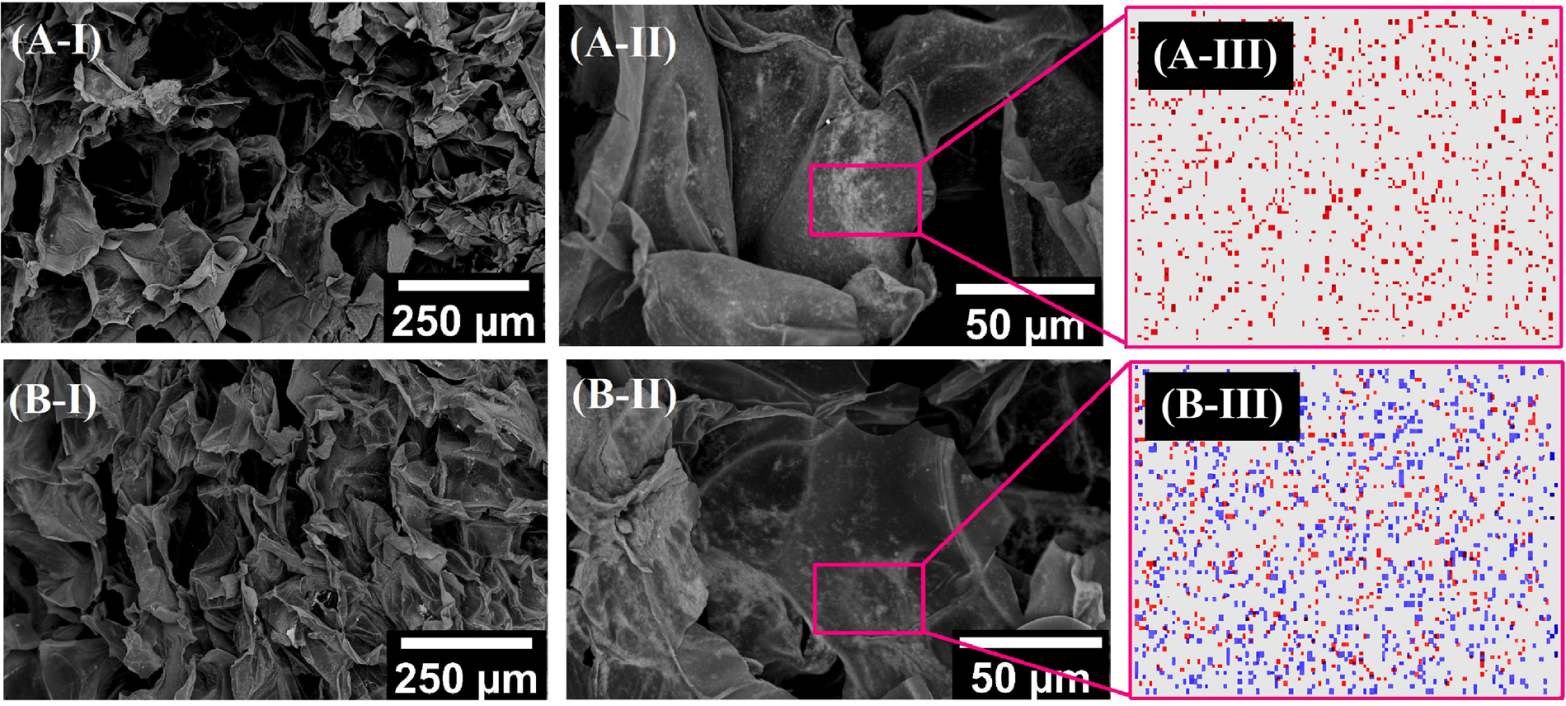
SEM micrographs and EDX mapping of lyophilized hydrogel samples: A—FeO_*x*_ filled HA hydrogel; B–FeO_*x*_:Al_2_O_3_ 1:1 filled HA hydrogel; I, II—SEM micrographs with different magnification; and III—EDX mapping, red and blue dots denote Fe and Al, respectively.

In order to be used as a selective heating agent the magnetic material should allow precise localization and uniform distribution within the desired tissue. In the current study, HA hydrogel was chosen as the matrix for MCPs due to its biocompatibility and ease of preparation^37^ and high porosity, as shown in the present case (see Fig. 3). This highly porous structure allows free diffusion of substances, thus creating an environment suitable for applications such as drug delivery (controlled release of therapeutics)^59^ or tissue engineering (exchange of nutrients and waste among the cells).^60,61^

EDX analysis confirmed uniform distribution of Fe in the case of the MCPs filled HA hydrogels (Fig. 3). This is a necessary condition for reproducibility of results obtained with the described material, and its subsequent applicability in the medical field. In the case of the addition of Al_2_O_3_ NPs to the MCPs dispersion, the EDX again showed the respective elements (Fe and Al) uniformly distributed throughout the material.

### D. Assessment of HA hydrogels as mediators of induction heating

**FIG. 4.**
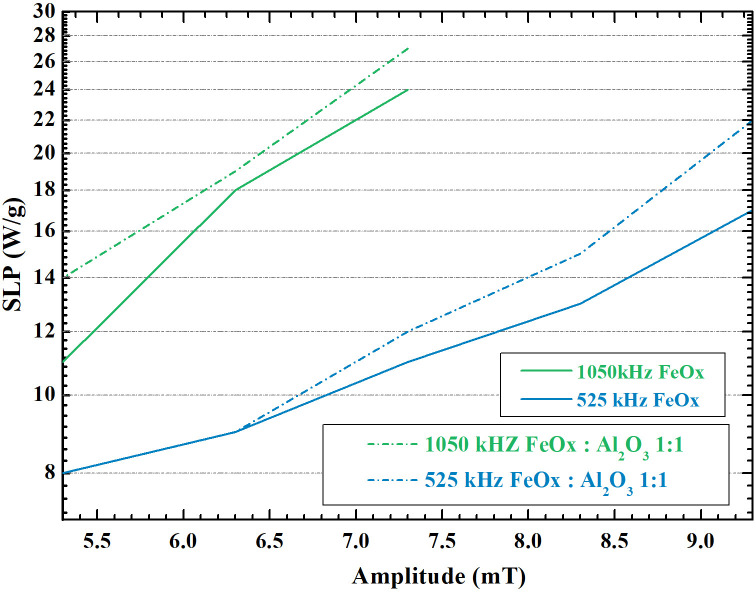
SLP of magneto-responsive HA hydrogel as a function of AMF amplitude.

The solid character of hydrogels is bound to restrain Brown relaxation and thus decreased SLP in a wide range of amplitudes of AMF (see Fig. 4). The hydrogels reached 25 W · g^−1^ as compared to aqueous dispersion reaching 40–60 W · g^−1^ in AMF of the frequency 1050 kHz and amplitude 7.4 mT. However, the SLP values in HA hydrogels are still higher compared to the values obtained in agar (15 W · g^−1^). Therefore, it is possible to assume that a certain portion of the heating is facilitated by Brown relaxation due to the softness of HA hydrogels allowing particle movement to some extent.^56^

The heating efficiency of the HA hydrogels filled with single MCPs or MCPs with Al_2_O_3_ NPs aggregates was determined in two frequencies of the AMF (525 and 1050 kHz) and the amplitudes ranging from 5.4 to 9.4 mT (Fig. 4). As can be seen, the hydrogels filled with MCPs and Al_2_O_3_ aggregates displayed higher heating ability regardless of the AMF parameters. This is in correspondence with the previously described experiments and confirms that Al_2_O_3_ shielding facilitates the Néel relaxation. The experimental results demonstrated that at AMF, 1050 kHz, 7.4 mT, considered safe for use in medicine,^21^ the samples heated at the rate of 0.3 °C per minute. Therefore, the hyperthermia temperature range could be reached within 16.7 min. Thus, the investigated magnetic HA hydrogels are promising materials for magnetic hyperthermia treatment, as well as other thermally triggered therapies.

### E. Rheology of the magneto-responsive hydrogel

**FIG. 5.**
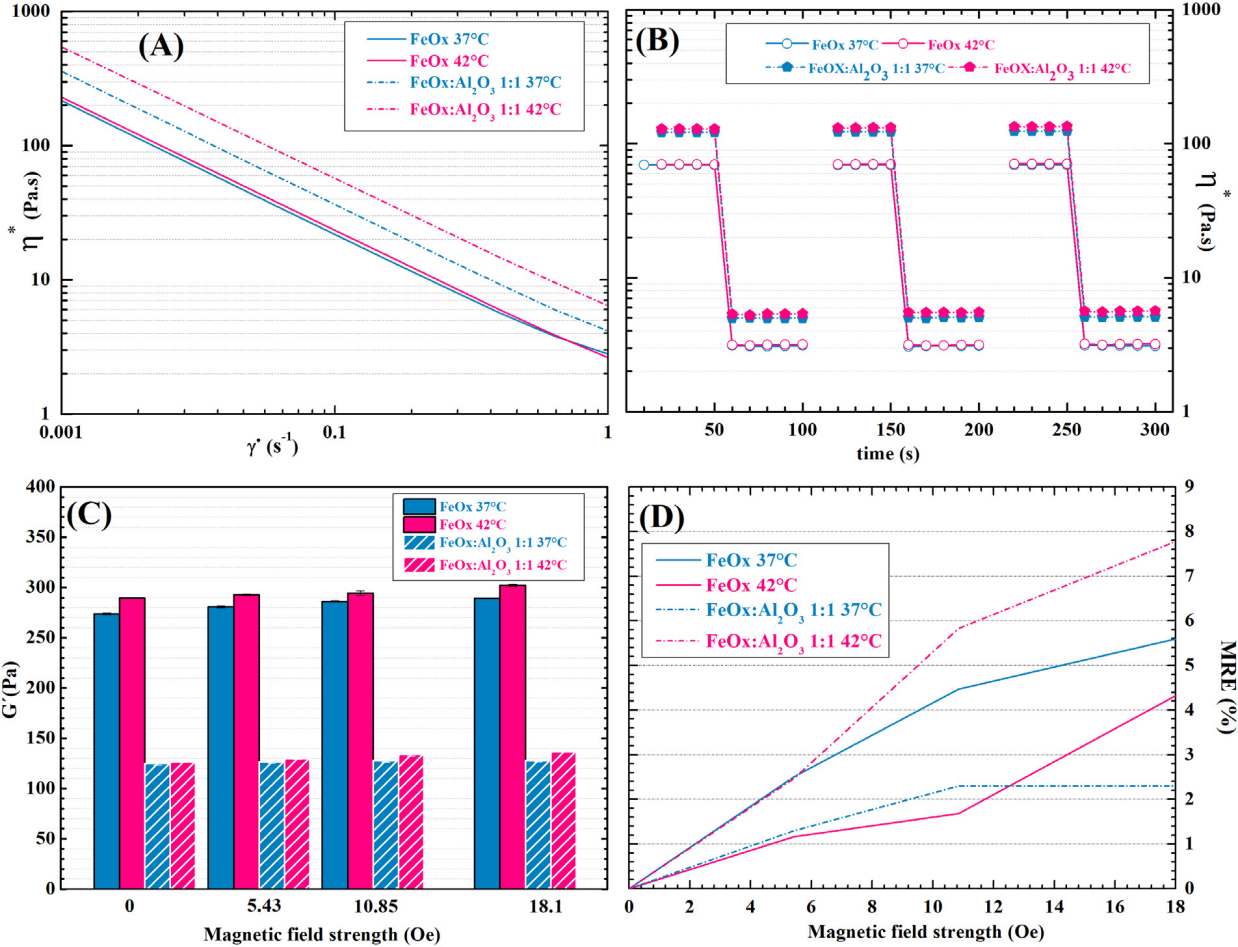
Rheological characterization of the magneto-responsive HA hydrogels: (A) dependence of magneto-responsive HA hydrogels viscosity on shear rate at body temperature (37 °C) and hyperthermia temperature (42°); (B) cyclic shear stress applied to hydrogels filled with FeO_*x*_ or FeO_*x*_:Al_2_O_3_ 1:1 at normal body temperature (37 °C) and at hyperthermia temperature (42 °C) respectively; (C) Storage moduli of magneto-responsive HA hydrogels at different temperatures in increasing external magnetic field; and (D) MRE induced in the magneto-responsive hydrogels by the external magnetic field.

The mechanical investigation of the hydrogels shows that increasing temperature from 37 °C (body temperature) to 42 °C (hyperthermia treatment) did not have any significant effect on hydrogels viscosity, or storage modulus [Figs. 5(A)–5(C)]. The hydrogels showed distinct shear thinning behavior, as their viscosity changes in the range of 10^2^ Pa · s in the shear rate range of 10^2^ s^−1^ [Fig. 5(A)]. The individual differences between the curves in Fig. 5(A) measured at different temperatures fall within the measurement error. Generally, shear-thinning hydrogels may find use as injectable, extrudable, and 3D printable materials.^62^ At the low shear rate the hydrogels demonstrate the solid-like behavior, i.e., the loss modulus does not exceed storage modulus, tan *δ* is lower than 1 [Figs. S1(a) and S1(b)].

Both hydrogels filled with MCPs and the mixture of MCPs and Al_2_O_3_ NPs aggregates displayed the storage modulus between 100 and 300 Pa [Fig. 5(C)], which corresponds to the values reported for normal fat tissue and spinal cord neural tissue.^38,39^ Therefore, the developed hydrogels can be for example useful in breast cancer treatment, as they would lower the discomfort of patients due to the close resemblance of the natural tissue.

The rheological measurements in the external magnetic field showed that the mechanical properties of the hydrogel are unaffected by the magnetic field, as the magneto-rheological effect (MRE) is less than 8% [Fig. 5(D)]. This is apparently due to the low size of magnetic MCPs (85 nm), as well as due to the low particle concentration (0.3 wt. %). This is another factor contributing to diminishing the patient's discomfort.

3D printing applications, in particular, require not only shear thinning behavior but also fast recovery due to the nature of the process, in which the high shear stress in the printhead is suddenly lifted upon placing on the printbed.^62^ As can be seen from Fig. 5(B), the hydrogels are capable of rapid change of rheological behavior in correspondence to the applied shear rate regardless of the temperature in the chosen temperature range. Additionally, the presence of Al_2_O_3_ has no observable impact on the reversibility of hydrogels' rheological behavior.

The cyclic shear stress testing proved the reversibility of Schiff base bonds forming the hydrogel. The material was able to return to the original viscosity and storage modulus from the fluid state within 30 s, as listed in Table I. These results make the proposed hydrogels promising materials for applications in extrusion-based 3D printing.^63^

**TABLE I.** Recovery of complex viscosity and storage modulus, respectively, during cyclic shear stress testing of magneto-responsive HA hydrogels.

	FeO_*x*_ (37 °C)		FeO_*x*_ (42 °C)	
	Recovery complex viscosity (%)		Recovery complex viscosity (%)	
	Low shear rate	High shear rate	Low shear rate	High shear rate
1. cycle	100	100	100	100
2. cycle	99.8	99.9	100.1	99.5
3. cycle	99.4	100.3	101	101.1
	Recovery storage modulus (%)		Recovery storage modulus (%)	
1. cycle	100	/	100	/
2. cycle	99.7	/	100.2	/
3. cycle	99.7	/	101.1	/
	FeO_*x*_:Al_2_O_3_ 1:1 (37 °C)		FeO_*x*_:Al_2_O_3_ 1:1 (42 °C)	
	Recovery complex viscosity (%)		Recovery complex viscosity (%)	
1. cycle	100	100	100	100
2. cycle	100.8	101.1	101.9	102.9
3. cycle	101.6	102.2	104.1	105.1
	Recovery Storage modulus (%)		Recovery Storage modulus (%)	
1. cycle	100	/	100	/
2. cycle	100.8	/	101.7	/
3. cycle	101.8	/	103.9	/

### F. Stability during cultivation and *in vitro* cytotoxicity of the hydrogels

**FIG. 6.**
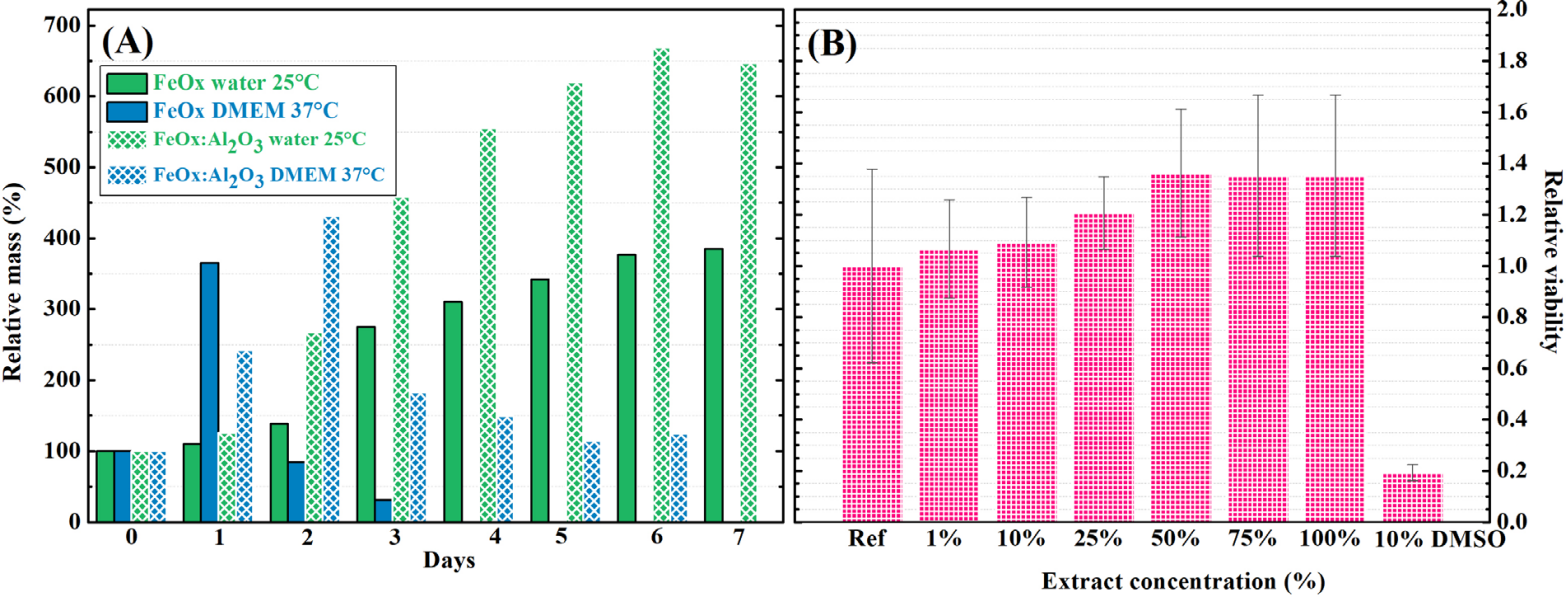
(A) Long-term stability of magneto-responsive HA hydrogels in water and simulated cultivation conditions; and (B) *in vitro* cytotoxicity evaluation of the hydrogels via extract method.

The proposed biomedical applications of the hydrogels, namely, magnetic hyperthermia, and neuromodulation, as well as tissue engineering, require stability of the hydrogel structure over the course of several days. Long-term stability of the HA hydrogels was tested in mild conditions (water at 25 °C), and in simulated cell cultivation conditions (complete DMEM at 37 °C) Fig. 6(A). The hydrogels were prone to significant solvent intake in water, reaching approximately 400% of their weight after 3 days in the case of Al_2_O_3_-containing samples, or 6 days for samples without Al_2_O_3_. Additionally, the hydrogels did not lose their structural integrity even after 7 days in water. In simulated cultivation conditions, the sample solvent intake is more rapid (approximately 400% with the relative deviation of 25% was reached in 1 or 2 days for samples without and with Al_2_O_3_, respectively). The stability is significantly decreased in the cell cultivation medium and increased temperature. The hydrogel containing only FeO_*x*_ was completely disintegrated within 3 days. However, the presence of Al_2_O_3_ in the hydrogel provided a stable structure for 6 days. Though there is a decrease in the relative mass for the hydrogel with Al_2_O_3_ after day 2, the experimental observations revealed that the hydrogel structure was kept until the 7th day. It is known that HA chains are greatly affected by the charges and ionic strength of their surrounding environment due to polyelectrolyte character.^64^ Thus, the particles' surface charge may significantly influence HA structures' stability. However, this effect would require further investigation in future studies. The long-term stability results revealed that the hydrogels containing Al_2_O_3_ are more suitable for biomedical applications.

The cytocompatibility of the HA hydrogel containing the MCPs and Al_2_O_3_ aggregates was examined by an indirect *in vitro* cytotoxicity test applying extract method [see Fig. 6(B)]. This method is useful for preliminary cytotoxicity screening. The hydrogel components are generally considered nontoxic. However, it is important to test their cumulative effect on cell viability as well. Extracts not causing a cell viability decrease below 70% are considered non-cytotoxic, as described in the standard ISO 10993-5. The relative cell viability increased slightly with the increase in the extract concentration. For all the extract concentrations it exceeds the 0.70 threshold value, thus proving the hydrogel cytocompatibility.

### G. Bioprinting

Bioprinting, i.e., 3D printing of material including living cells, allows a homogeneous distribution of cells throughout the construct, increasing the usability in tissue engineering.^45^ Nevertheless, using the bioprinting technique in hyperthermia research would provide an opportunity to fabricate precise tumor tissue analogs and potentially aid the research and use of hyperthermia as a complementary therapeutic procedure in clinical practice.^65,66^ The selected polymer matrix, HA, with a molecular weight below 10^6^ Da, was observed to promote tumorigenesis in breast cancer, which is further advantageous in cultivating artificial tumor tissue.^67^

Materials for bioprinting, commonly known as bio-inks, need to be carefully tailored to facilitate sufficient printability on the one hand and cell viability and biological functionality on the other. The bioprinting process exposes the printed material to significant shear stress due to forced flow through a narrow needle.^68^ The bio-ink matrix should therefore undergo the transition from high viscosity to low viscosity in the range of shear rate exposed in typical extrusion-based 3D printing to minimize the shear stress imposed on cells encapsulated in the material and simultaneously maximize the cell viability.^69^ The shear thinning behavior of the proposed hydrogels, as was demonstrated earlier in III E, clearly shows that the material complies with the first condition for viable bio-ink.

The requirement of low shear stress during extrusion would encourage using low-viscosity materials in general. However, such materials fail to provide sufficient printing precision and shape fidelity due to the spreading of the material upon placing it on the printbed.^62^ Therefore, rapid recovery of the material, as was demonstrated by cyclic shear stress measurement [Fig. 5(B)], is necessary to facilitate sufficient precision of the printed structure.

The biological functionality condition mainly depends on the desired tissue type in terms of mechanical, chemical, and morphological points of view. The soft and pliable materials developed in this study are mechanically in the range of soft tissue types, typically adipose tissue^38^ or spinal cord neural tissue.^39^ Furthermore, the magnetic heating ability provided by the MCPs would enable heat-triggered neuromodulation, making the material promising beyond the scope of hyperthermia, advancing toward tissue engineering and regenerative medicine of neural tissue.

**FIG. 7.**
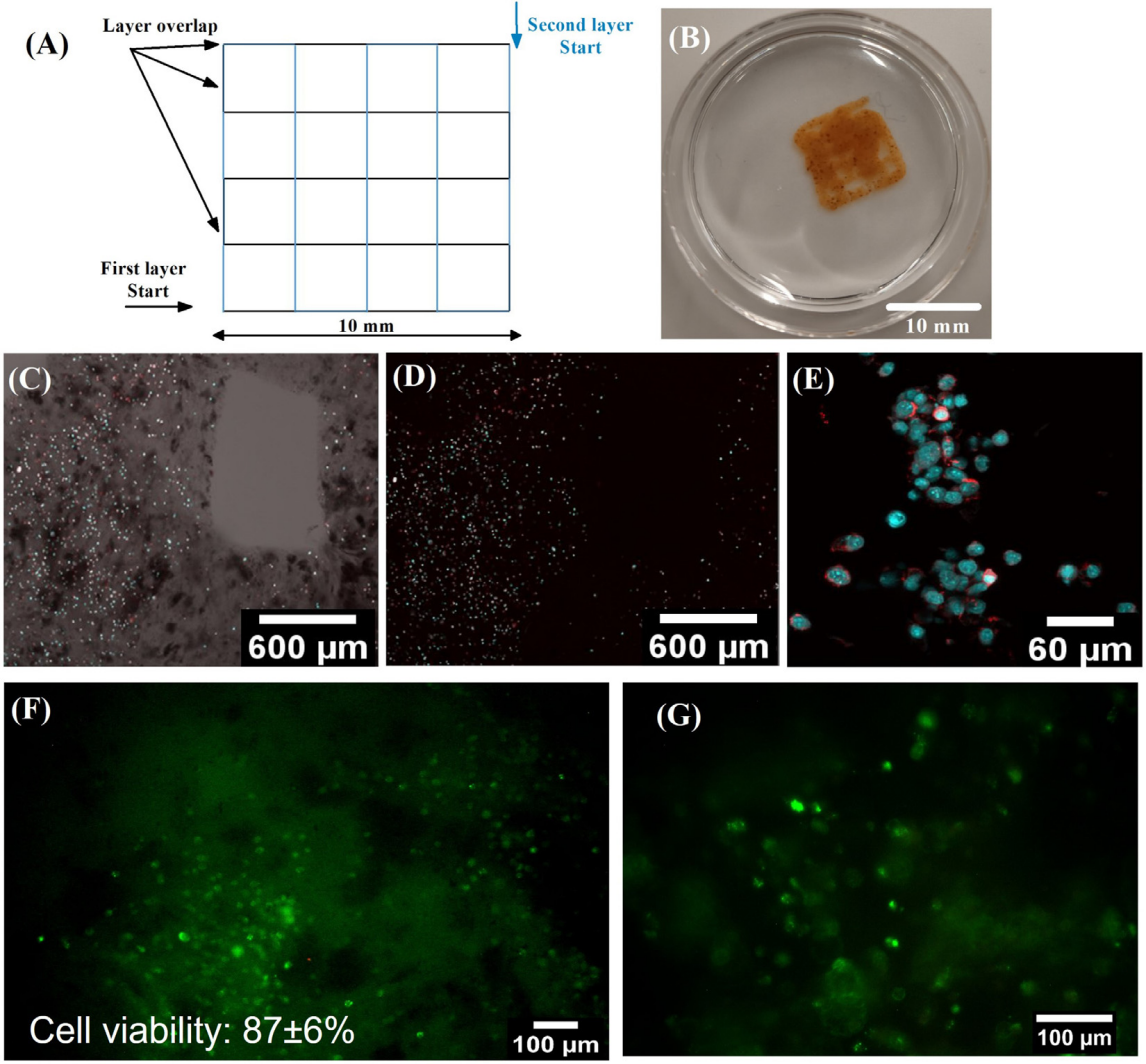
Bioprinting of BALB/3T3 mouse fibroblasts encapsulated in magneto-responsive HA hydrogel: (A) printing model; (B) printed grid photography taken on the eighth day after printing; (C)–(E) confocal imaging of cells distribution within the scaffold in bright field (C) and fluorescence channel (C)–(E). Cells were stained for cytoskeleton (red) and nucleus (blue); (F) and (G) live/dead assay of BALB/3T3 fibroblasts. The cells were visualized in fluorescence immediately after the microextrusion.

The bioprinting of the bio-inks prepared by encapsulation of BALB/3T3 mouse fibroblasts in HA hydrogels containing FeO_*x*_ MCPs with Al_2_O_3_ aggregates was done with sufficient printing precision and cell distribution homogeneity [Figs. 7(A)–7(E)]. Additionally, the live/dead staining proves that the cells retained their metabolic activity after printing [Figs. 7(F) and 7(G)], and there was no detectable damage to the cell membranes, as 87± 6% cell viability was achieved. Therefore, the materials reported here have been successfully tested as bio-inks for extrusion-based 3D bioprinting.

## III. CONCLUSION

The magneto-responsive hydrogel composed of HA matrix cross-linked via Schiff base formation, magnetic MCPs, and aluminum oxide NPs aggregates was elaborated. The MCPs ensure the hydrogel heating under exposure to an AMF with parameters approved for medicine. It was shown that the presence of Al_2_O_3_ NPs aggregates primarily influenced the Néel relaxation of the MCPs, while the Brown relaxation remained intact. On the base of the experimental findings, it can be concluded that the diamagnetic Al_2_O_3_ NPs aggregates apparently, shield the magnetic interactions among the MCPs, causing the increase in Néel relaxation and heating efficiency. A heating rate of 0.3 °C per minute was observed. The rheological measurements of the HA hydrogel confirmed the shear-thinning behavior and fast recovery after high shear stress. Additionally, the presence of Al_2_O_3_ NPs aggregates increases the long-term stability of the hydrogel at cell cultivation conditions, making them more suitable for tissue engineering-related applications. The hydrogel was proven to be cytocompatible. Moreover, it was demonstrated that viscoelastic properties of the hydrogel allowed extrusion-based 3D bioprinting with BALB/3T3 mouse fibroblasts. Self-supported multi-layered uniformly porous structure was formed with homogeneous cell distribution and high cell viability. The combination of high heating efficiency, softness, biocompatibility, and 3D printability of magnetic HA hydrogel allows to consider it a multifunctional material suitable for AMF induced heating in biomedical applications such as magnetic hyperthermia,^6,28^ wireless thermal brain stimulation,^12,13,70,71^ thermally triggered drug delivery^7,10,30^ as well as for precise scaffold engineering and bioprinting.^1,72,73^

## IV. METHODS

### A. MCPs synthesis

FeO_*x*_ NPs were prepared by co-precipitation of FeCl_2_ (in the form of FeCl_2_ · 2 H_2_O, Merck KGaA, Germany) and FeCl_3_ (in the form of FeCl_3_ · 6 H_2_O, Merck KGaA, Germany) in alkaline solution according to the procedure described previously.^24^ Briefly, the respective salts in Fe^2+^:Fe^3+^ 1:2 molar ratio were dissolved in demineralized water (Millipore Q System, Millipore, UK), and added dropwise to 0.38 M NH_3_ solution (diluted from NH_3_ 30%, Penta, Czechia) at 70 °C. The reaction was carried on for 1 h under vigorous stirring, 700 rpm. After that, a dark brown precipitate was obtained, containing FeO_*x*_ NPs of 13 nm and polydispersity 0.3 according to transmission electron microscopy (TEM).^24^

To achieve peptization and size separation of the FeO_*x*_ MCPs, a procedure described in Smolková *et al.*^47^ was followed. The procedure requires washing of the FeO_*x*_ NPs precipitate 3 times with demineralized water, followed by acidification with 1 mM HCl (diluted from 35% HCl, Penta, Czechia) to pH 2,5 and ultrasonication for 20 min. After that, the dispersion was let to sediment on a strong permanent magnet for 30 min, and the brown supernatant was collected. The dispersion of particles collected at pH 2,5 with the average particle size 85 nm and zeta potential 45–55 mV^47^ was used in the study. The concentration of FeO_*x*_ in the dispersion was determined by x-ray fluorescence spectroscopy using ARL Quant'X EDXRF Analyzer (Thermo Scientific, MA, USA). The Al_2_O_3_ nanopowder (13 nm primary particle size, 99.8% trace metal basis; Merck KGaA, Germany) was mixed with FeO_*x*_ by adding the appropriate amount of Al_2_O_3_ powder to the FeO_*x*_ dispersion to obtain weight ratios of FeO_*x*_:Al_2_O_3_ 4:1, 3:1, 2:1, 1:1, and 1:2, respectively. Then, the mixtures were sonicated for 20 min.

### B. Particle size and zeta potential analysis

The hydrodynamic size of MCPs and zeta potential were measured by dynamic light scattering (DLS) and laser Doppler velocimetry on Zetasizer Nano ZS (Malvern Panalytical, UK). The hydrodynamic radii of particles, expressed as z-average particle diameters, were measured at 25 °C at a scattering angle of 173°. The polydispersity index (PDI) describing the width of the particle size distribution in a given sample was also determined. The measurements were performed in triplicate, and an arithmetic average of the results is presented.

### C. HA hydrogels preparation

HA cross-linking via Schiff base formation was performed with two polysaccharide derivatives—adipic acid dihydrazide (ADH) grafted HA (HA-ADH), and oxidized dextran (DEX-OX). The derivatization and hydrogel formation is thoroughly described in Vítková *et al.*^37^ and Musilová *et al.*^48^ Briefly, HA-ADH was prepared by 4–(4,6-Dimethoxy-1,3,5-triazin-2-yl)-4-methylmorpholinium chloride (DMTMM; Merck KGaA, Germany) mediated reaction. HA (243 kDa; Contipro, Czechia) 1 wt. % solution was obtained by dissolving the polymer in demineralized water at 50 °C. After cooling to room temperature, DMTMM and ADH (Merck KGaA, Germany) was added to the reaction mixture. The molar ratio of the reactants HA:DMTMM:ADH was 4:1:4. The reaction proceeded for 24 h at 25 °C under constant stirring. DEX-OX was, on the other hand, prepared by periodate oxidation. The procedure was derived from Maia *et al.*^49^ Dextran (40 kDa, Merck KGaA, Germany) was dissolved in demineralized water by mixing at 50 °C overnight to obtain a 13 wt. % solution. After cooling the solution to room temperature, sodium periodate (Merck, NJ, USA) was added in a 5:2 molar ratio of dextran:NaIO_4_. The reaction was left to proceed under constant mixing for 4 h at 25 °C in the dark. In both cases, the products were purified by dialysis against distilled water for three days (cut-off 5000 Da) and subsequently frozen and lyophilized.

To produce hydrogels, HA-ADH and DEX-OX were dissolved in MCPs dispersions (without or with Al_2_O_3_) by shaking overnight and at 25 °C to obtain 2 wt. % of modified polymer in the solution. Afterward, the solutions of HA-ADH and HA-OX were mixed by vortex, and Schiff-base formation occurred spontaneously. The gelation process was finished within 30 min after the mixing.

### D. Induction heating in AMF

The heating efficiency of the prepared MCPs in three different forms was determined: water dispersions, high-viscosity agar dispersions, and Schiff base cross-linked HA hydrogels. To obtain the dispersions of MCPs in the agar matrix, 3.4 wt. % of agarose (Merck KGaA, Germany) was added to the water dispersion of MCPs and heated under continuous stirring to 70 °C. After this, the mixture was placed in a fridge, where the solid matrix was quickly formed. The samples of HA hydrogel were prepared as described in Sec. II C, and left to gelate at 25 °C for at least 1 h to ensure full cross-linking of the mixture.

A homemade AMF generator was used to determine the heating efficiency of the samples. It consisted of a signal generator Agilent 33521A (Agilent Technologies, CA, USA), RF broadband amplifier AR RF/Microwave Instrumentation 800A3A, induction coil (90 mm diameter), interchangeable capacitors, and magnetic field sensor. The measurements were carried out at AMF of 525 kHz frequency and amplitude range 5.4–9.4 mT, or 1050 kHz frequency and amplitude range 5.4–7.4 mT, respectively. The temperature was measured with monitoring system ReFlex 4, Neoptix (Qualitrol, NY, USA), and fiber optic temperature sensor T1S-03-PT06 inserted directly in the sample.

### E. Morphological and elemental analysis

The inner morphology of the hydrogels was examined by scanning electron microscopy (SEM) of cross sections of lyophilized samples of hydrogels using Phenom XL G2 instrument (Thermo Scientific, MA, USA). The 3D printed hydrogel samples were frozen first at −18 °C for 24 h, followed by freeze drying in a freeze-dryer (ALPHA1–2 LD plus, M. Christ, Osterode am Harz, Germany). Additionally, Phenom XL G2 instrument (Thermo Scientific, MA, USA) was used for elemental analysis by energy-dispersive x-ray spectroscopy (EDX). The accelerating voltage used for analysis was 15 kV. The samples were sputtered with a gold/palladium layer before measurement.

### F. Rheological analysis

Rheological measurements were performed using a rotational rheometer, Anton Paar MCR 502 (Anton Paar, Austria), at human body temperature (37 °C) and hyperthermia temperature (42 °C) under normal pressure in air, using an MRD 180/1T measuring cell with parallel plate 20/MRD/TI measuring geometry in oscillation at constant deformation (varying to attribute to the linear region) with shear rate increasing from 0.001 to 1 s^−1^. The hydrogel samples were prepared 1 hour in advance in the form of circular plates with a diameter of 25 mm and a thickness of 1.5 mm. Viscosity is shown with respect to the shear rate in the measured range, i.e., from 0.001 to 1 s^−1^. On the other hand, storage modulus (G') is expressed as the mean value measured in the range 0.01–0.1 rad · s^−1^, which corresponds to the linear region with a constant value of G′. Additionally, cyclic oscillatory shear stress was applied to the samples in the following specifications: constant low (0.03 s^−1^) and high shear rate (0.8 s^−1^) were applied for 50 s. The low-high shear rate cycle was repeated 3 times. The deformation was kept constant throughout this measurement.

### G. Long-term stability and *in vitro* cytotoxicity

To asses the long-term stability of the hydrogels, the samples were placed in perforated sample holders and submerged in desired medium and conditions, either demineralized water at 25 °C or Dulbecco's Modified Eagle's Medium (PAA Laboratories GmbH, Austria) containing 10% bovine calf serum (BioSera, France) and 1% of Penicillin/Streptomycin (GE Healthcare HyClone, United Kingdom), hereinafter denoted “complete DMEM” for simplification, at 37 °C. The samples were weighted once every 24 h. The results are expressed as the relative changes in weight, where the original sample weight is considered 100%. The measurement was performed in triplicate, and the average results are given here.

Cytotoxicity testing was done according to ISO standard 10993 using a mouse embryonic fibroblast cell line (ATCC CRL-1658 NIH/3T3, USA). The complete DMEM was used as a cultivation medium. The cell line was incubated at 37 °C in 5% CO_2_ in humidified air. The relative humidity value during the cell line incubation was 95%. Sterilization of hydrogels was done before cytotoxicity testing by shaking samples in 70% ethanol for 1 h at laboratory temperature. Elution of the ethanol from samples was done by aspiration, a short rinsing in PBS, and then by shaking the samples in ultrapure water for 48 h at laboratory temperature. After ultrapure water aspiration, extracts were created. Extracts were prepared according to ISO standard 10993-12 (100 mg of hydrogel per 1 ml of media). The tested material was incubated in a cultivation medium for 24 h at 37 °C with stirring. The parent extracts (100 vol. %) were then diluted in a medium to obtain a series of dilutions with concentrations of 75, 50, 25, 10, and 1 vol. %. All extracts were used for up to 24 h. The cells were seeded at a concentration of 10^5^ per well in 96-well plates (TPP, Switzerland). After the pre-incubation period (24 h), the extracts were filtered using a syringe filter with a membrane pore size of 0.22 *μ*m (TPP, Switzerland) to ensure that no residual hydrogel was present. The filtered extracts in required dilutions were added to the cells and incubated for 24 h. Subsequently, tetrazolium salt (MTT cell proliferation assay kit, Duchefa Biochemie, Netherlands) was used to determine cell viability. Absorbance was measured using a microplate reader Infinite M200 PRO (Tecan, Switzerland) at 570 nm, and the reference wavelength was adjusted to 690 nm. The results are presented as the percent reduction of cell viability when compared to cells cultivated in a medium without the extracts of tested materials. The morphology of cells from the culture plates was observed using an inverted Olympus IX 81 phase-contrast microscope (Olympus, Japan).

### H. Bioprinting

Bioprinting was done with BALB/3T3 mouse fibroblasts cell line. The cells were cultivated in complete DMEM at 37 °C in a humidified incubator (95% relative humidity, 5% CO_2_, Heracell, Germany). The cells were split using 0.05% trypsin (Merck KGaA, Germany).

Bioinks were obtained by adding 100 *μ*l of cell suspension in PBS (total amount of BALB/3T3 was 3 × 10^6^) into 1 ml of immediately mixed HA-ADH and DEX-OX solutions in FeO_*x*_:Al_2_O_3_ dispersion. The encapsulation of the cells within the bio-ink matrix was achieved by rapid gelation of the polymer precursors. The bio-inks were loaded into cartridges and printed by pneumatic extrusion printhead. To this end, 3D Discovery Bioplotter (RegenHU, Switzerland) was used. The printing was done through a cylindrical needle of 0.52 mm inner diameter. The printing model was chosen as a simple 1 × 1 cm^2^ grid. The thickness of each layer was 0.5 mm. The scaffold consisted of two layers and had a thickness of 1 mm. The printing pressure was 2.078 × 10^5^ Pa.

Fluorescence imaging of the distribution of the cells through the material was done by means of Laser Scanning Confocal Microscopy (LSCM) using the Olympus FLUOVIEW FV3000 (Olympus, Japan) device. The Plan-Apochromat objective with magnification 10× and numerical aperture NA = 0.8, or 4× and NA = 0.4, respectively, were used for analysis. The figures were obtained as three-dimensional reconstructions from confocal images in the z-axis. The cell-loaded samples were fixed and fluorescence stained following this protocol: the staining solution containing Phalloidin DyLight 488 (Thermo Fisher Scientific, Germany) and DAPI (Thermo Fisher Scientific, Germany), each in 1:1000 dilution in PBS, was prepared immediately before fixing and kept in the dark. The samples were washed 3 times with PBS. After that, the cells were fixed with 3.7% formaldehyde (Thermo Fisher Scientific, Germany) solution for 25 min at room temperature. The fixative solution was aspirated, and the samples were washed once with PBS. The samples were then submerged in 0.1% Triton X-100 (Thermo Fisher Scientific, Germany) solution for 30 min to permeabilize cell membranes. After aspiration of permeabilization solution and washing samples once with PBS, the samples were submerged in the staining solution for 1 h. Finally, the samples were washed twice with PBS and kept in a dark, cold, and humid environment before fluorescence imaging.

Directly following 3D bioprinting, live/dead staining was performed. The staining solution was prepared as follows: 2 *μ*l of ethidium homodimer I (dead stain; Thermo Fisher Scientific, Germany) and 2 *μ*l calcein acetoxymethyl ester (live stain; Thermo Fisher Scientific, Germany) were diluted in 10 ml of PBS. The staining solution was poured over the prints in sufficient amount, and left in an incubator for 1 h to allow full diffusion of the staining solution through the material. The stained cells were observed in a LifeCell fluorescence microscope (DMI6000, Leica, Wetzlar, Germany). The percentage of live cells was determined as an average of images obtained from 3 different spots in the sample.

### I. Statistical evaluation

Where appropriate, respective standard deviations of the arithmetic mean for the 68.3% confidence interval are presented along with the arithmetic mean value. The p-value of 0.05 was used when appropriate.

## SUPPLEMENTARY MATERIAL

See the supplementary material includes the rheological properties of HA hydrogels, namely, the dependence of the storage modulus and loss modulus on the shear rate, and the dependence of tan *δ* on the shear rate (Fig. S1), as well as the magnetization curves of FeO_*x*_ MCPs powder and Al_2_O_3_ NPs (Fig. S2).

## Data Availability

The data that support the findings of this study are available from the corresponding author upon reasonable request.
